# Comparative analysis of GCF β-glucuronidase level in diabetic and nondiabetic patients with chronic periodontitis: A clinicobiochemical study

**DOI:** 10.4103/0972-124X.44092

**Published:** 2008

**Authors:** Aarti Chowdhary, G. V. Gayathri, Dhoom Singh Mehta

**Affiliations:** 1 *Former Post graduate Student, Department of Periodontology and Implantology, Bapuji Dental College and Hospital, Davanagere – 577 004, Karnataka, India*; 2 *Professor, Department of Periodontology and Implantology, Bapuji Dental College and Hospital, Davanagere – 577 004, Karnataka, India*; 3 *Professor and Head, Department of Periodontology and Implantology, Bapuji Dental College and Hospital, Davanagere – 577 004, Karnataka, India*

**Keywords:** β - Glucuronidase, chronic periodontitis, diabetes, GCF

## Abstract

Recent evidences prove that, release of potent lysosomal enzymes e.g. β-Glucuronidase by degranulation of polymorponuclear leukocytes in host gingiva may contribute significantly to tissue destruction and the pathogenesis of periodontal disease. The purpose of the present study was to compare and correlate GCF β-Glucuronidase with periodontal status among diabetic and non-diabetic patients with chronic periodontitis. A total number of 75 patients were equally divided into Group I (control group), Group II (non diabetic with chronic periodontitis) and Group III (diabetic with chronic periodontitis). Clinical parameters like Plaque index, Gingival index, Probing Pocket Depth and RBS were recorded. The β-Glucuronidase level in GCF of all three groups was determined by spectrophotometric analysis. It was observed that the periodontitis patients irrespective of their diabetic status, showed increased periodontal destruction with elevated level of β-Glucuronidase than the controls. Also, the diabetic patients showed increased severity of periodontal destruction and the elevated level of β-Glucuronidase, thus indicating diabetics at a higher risk for progressive periodontal destruction.

## INTRODUCTION

Much effort has been made in recent years to identify risk factors responsible for initiation and progression of periodontal diseases. Mounting evidences indicate that, gingivitis and periodontitis are caused by various host responses which are associated with the continuous presence of microorganisms in the gingival crevice.[1] They may cause disease either by decreasing the host defense capability or by triggering a variety of local inflammatory responses. Increasingly, attention has been directed to assaying various byproducts of the host bacterial interactions like Aspartate Aminotransferase, Lactate Dehydrogenase, Arylsulphatase, Cathepsins, β-Glucuronidase etc.

Gingival Crevicular Fluid (GCF) provides a non invasive means of studying the host response factor by change of constituents in the fluid. The inflammatory exudates from gingival microcirculation crosses inflamed periodontal tissue and en route collects molecules of potential interest from the local inflammatory reaction. Therefore, the fluid offers a great potential source of factors like enzymes that may be associated with tissue destruction.[2]

β-Glucuronidase is a neutrophil derived lysosomal acid hydrolase enzyme which is stored in the neutrophil primary (azurophil) granules and is released in response to inflammation during periodontal destruction. This enzyme is active in degradation of proteoglycans and the ground substance and is an indicator of neutrophil (PMN) influx into the crevicular environment. Lamster[3] in a recent study, observed that β-glucuronidase in GCF correlated well with clinical parameters like Plaque Index, Gingival Index, Bleeding Index, Probing Pocket Depth and Clinical Attachment Level of individuals sites. This enzyme also proved to be a good predictor of the response to treatment and the risk for future periodontal breakdown.

Periodontal disease has been shown to be more severe in diabetics as compared to non-diabetics.[4] Significant progressive destruction of periodontal apparatus occurs in diabetics as compared to non-diabetics and significantly higher level of β-Glucuronidase levels exists in patients with poorer diabetic control.[5]

Although, a positive relationship of β-Glucuronidase level and traditional clinical parameters for diagnosis of periodontal disease has been widely studied, but there are inadequate number of studies for comparing β-Glucuronidase level as a potential marker for disease activity in diabetic and non diabetic patients suffering form chronic periodontitis. Hence, the present study was conducted with the following aims and objectives:

- To compare GCF β-glucuronidase activity with periodontal status among diabetic and non-diabetic patients.
- To correlate GCF β-glucuronidase activity with periodontal status among diabetic and non-diabetic patients.

## MATERIALS AND METHODS

The patients for this study were selected from the Department of Periodontology and Implantology, Bapuji Dental College and Hospital, Davangere, Karnataka State.

### Inclusion criteria

1. Patients with good oral hygiene and clinically healthy periodontium (Group I).
2. Non-diabetic patients having probing pocket depth of 5-9 mm with a radiographic evidence of bone loss on at least 2 teeth, in minimum of two quadrants (Group II).
3. Diabetic patients having probing pocket depth of 5-9 mm with a radiographic evidence of bone loss (Group III) on at least 2 teeth, in minimum of two quadrants.

### Exclusion criteria

1. Patients with history of intake of any antibiotics and regular use of anti-inflammatory medication or the use of other drugs known to affect the periodontium, in past 6 months.
2. Patients who had professional teeth cleaning or any other periodontal treatment within last 6 months.
3. Patients who were unable to perform routine oral hygiene procedures.

### Study design

A total number of 75 patients, (43 females and 32 males) in the age group of 30-60 years were selected for this study. The selected patients were divided into three groups as follows:

Group I: (Control Group) consists of 25 patients with clinically healthy periodontium and good gingival status (< 25% of gingival index).

Group II: Consists of 25 non-diabetic patients with chronic periodontitis having 5-9 mm probing pocket depth and radiographic evidence of bone loss.

Group III: Consists of 25 diabetic patients with chronic periodontitis having 5-9 mm probing pocket depth and radiographic evidence of bone loss.

### Clinical parameters

The following clinical parameters were recorded after the selection of test sites.

1. Plaque Index (PI) (Silness and Loe 1964)
2. Gingival Index (GI) (Loe and Silness 1963)
3. Probing Pocket Depth (PD) was measured by using William's graduated periodontal probe, held parallel to the long axis of tooth from free gingival margin to base of pocket.
4. Random blood sugar (RBS) level was recorded for Group II and Group III patients.

### Principle

The rate of hydrolysis of phenolphthalein glucuronidase serves to assay the activity of β-Glucuronidase.

$$\mathrm{Phenolphthalein mono-\beta -Glucuronic acid}\overset{\mathrm{\beta -Glucuronidase}}{\to }\mathrm{Phenolphthalein}\;+\;\mathrm{Glucuronic Acid (Pink in Alkali)}$$

The phenolphthalein liberated is estimated by the red colour which it gives, at alkaline pH. Phenolphthalein glucuronide has hardly any absorption at the same pH.[6]

**Figure 1:**
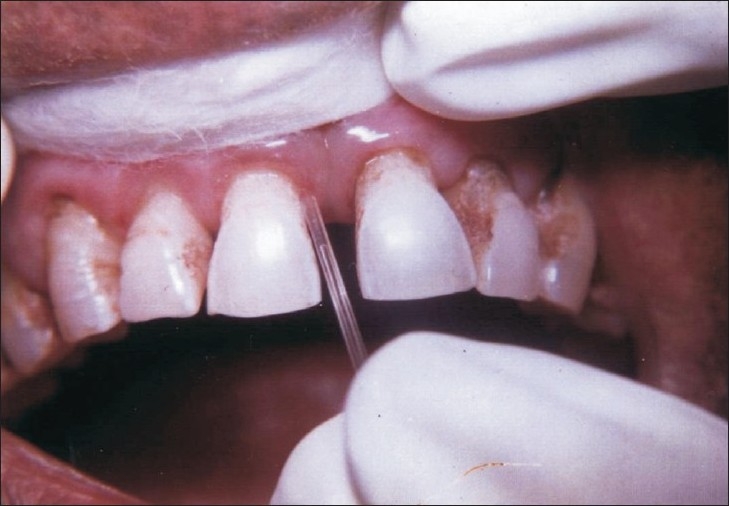
Collection of GCF through micropipette

A single test site was selected from each patient of Group I, II and III. After light supragingival scaling, a standard volume of 0.5 µl GCF was collected extrasulcularly from isolated test site [Figure 1]. Collected GCF was immediately transferred to 200 micro liter of normal saline and sent to the laboratory for analysis.

### Estimation of β-Glucuronidase (BG) level in GCF:

**Figure 2:**
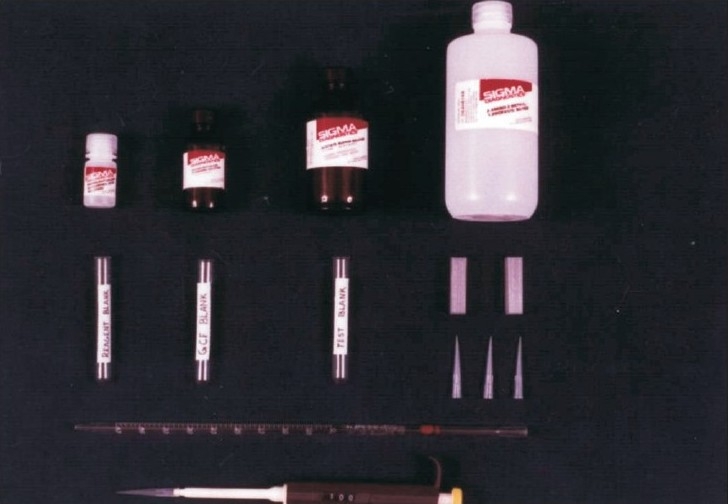
Signa reagent kit

**Figure 3:**
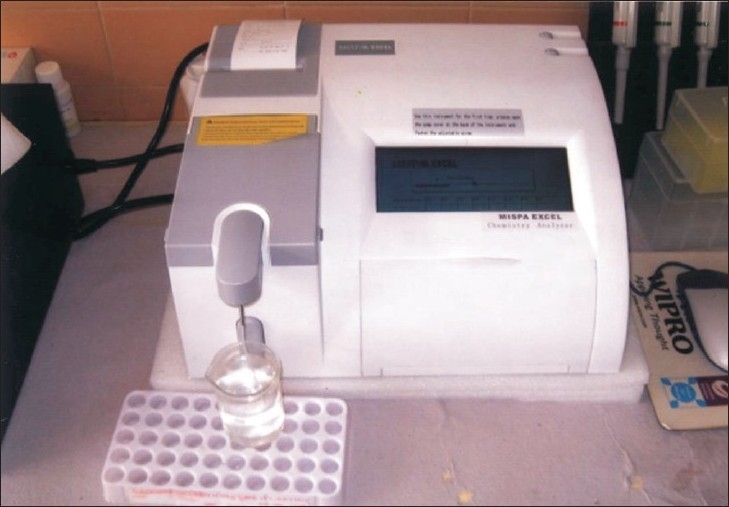
ERBA cherm pro semi auto analyzer

The β-Glucuronidase level in GCF was estimated by using the reagent kit supplied by Sigma Diagnostics [Figure 2] and spectrophotometric analysis was done by using ultraviolet-spectrophotometric method using a ERBA Chem Pro Semi Auto Analyser [Figure 3]. The method used, was based upon the modified procedure of Fishman *et al*.[6]

## RESULTS

Between the study groups, a significant increase in PI, GI, PD, RBS and β-Glucuronidase level was observed [Table 1]. On comparison between group I and II and between group I and III, a significant increase in PI, GI, PD and β-Glucuronidase level was observed in Group II and III [Table 2 and 3]. On comparison between group II and III, a significant increase in PD, RBS and β-Glucuronidase level even with non significant PI, GI score was observed in Group III [Table 4]. On correlation of β-Glucuronidase level with various clinical parameters, a significant correlation was observed only for PD in Group II and for PD and RBS in Group III [Table 5].

**Table 1:** Plaque index, gingival index, probing depth, random blood sugar and β-Glucuronidase levels in group I, II and III

Groups	N	Particulars	Plaque index	Gingival index	Probing depth	Random blood	β-Glucuronidase
Group I	25	Range	0.22 − 1.24	0.20 − 1.30	1.01 − 2.35	-	1.25 − 24.5
		Mean	0.68	0.66	1.45	-	7.03
		SD	0.36	0.37	0.36	-	5.73
Group II	25	Range	0.40 − 2.48	0.61 − 2.66	2.06 − 4.96	58 − 124	6.0 − 49.8
		Mean	1.38	1.45	3.50	85.6	25.39
		SD	0.50	0.47	0.69	16.1	11.96
Group III	25	Range	0.76 − 2.13	0.72 − 2.38	2.85 − 5.78	98 − 309	6.5 − 74.1
		Mean	1.36	1.49	4.03	140.9	33.33
		SD	0.39	0.39	0.77	46.2	14.78
Difference between groups		F-value*	22.6	31.6	11.5	32.0	34.6
		P-value	<.001	<.001	<.001	<.001	<.001

* One Factor ANOVA, *P*<.001 HS

**Table 2:** Comparison of plaque index, gingival index, probing depth, random blood sugar and β-Glucuronidase level between group II and group II

Parameter	Group I		Group II		t-value*	*P*-value	Significance
					
	Mean	SD	Mean	SD			
Plaque index	0.68	0.36	1.38	0.50	5.700	<.001	HS
Gingival index	0.66	0.37	1.45	0.47	6.58	<.001	HS
Probing depth	1.45	0.36	3.50	0.69	13.06	<.001	HS
RBS	-	-	85.6	16.1	-	-	-
β-Glucuronidase	7.03	5.73	25.39	11.96	6.92	<.001	HS

* Student's t-test, *P*<.05 significant, *P*<.001: Highly significant, *P*>.05: Not significant, RBS: Random blood sugar.

**Table 3:** Comparison of plaque index, gingival index, probing depth, random blood sugar and β-Glucuronidase level between group I and group III

Parameter	Group I		Group III		t-value*	*P*-value	Significance
					
	Mean	SD	Mean	SD			
Plaque index	0.68	0.36	1.36	0.39	6.42	<.001	HS
Gingival index	0.66	0.37	1.49	0.39	7.63	<.001	HS
Probing depth	1.45	0.36	4.03	0.77	15.21	<.001	HS
RBS	-	-	14.09	46.2	-	-	-
β-Glucuronidase	7.03	5.73	33.33	14.78	8.29	<.001	HS

* Student's t-test, *P*<.05 Significant, *P*<.001: Highly significant, *P*>.05: Not significant, RBS: Random blood sugar.

**Table 4:** Comparison of plaque index, gingival index, probing depth, random blood sugar and β-Glucuronidase level between group II and group III

Parameter	Group II		Group III		t-value*	*P*-value	Significance
					
	Mean	SD	Mean	SD			
Plaque index	1.38	0.50	1.36	0.39	0.15	0.88	NS
Gingival index	0.66	0.37	1.49	0.39	0.28	0.78	NS
Probing depth	3.50	0.69	4.03	0.77	2.57	<0.05	Sig
RBS	85.6	16.1	140.9	46.2	5.65	<.001	HS
β-Glucuronidase	25.39	11.96	33.33	14.78	2.09	<.05	Sig

* Student's t-test, *P*<.05 Significant, *P*<.001: Highly significant, *P*>.05: Not significant, RBS: Random blood sugar.

**Table 5:** Correlation of β-Glucuronidase level with plaque index, gingival index, probing depth and random blood sugar in group I, II and III

Variable	Group I		Group II		Group III	
			
	β - Glucuronidase	Significance	β - Glucuronidase	Significance	β - Glucuronidase	Significance
Plaque index	− 0.06	NS	− 0.01	NS	− 0.08	NS
Gingival index	0.08	NS	0.16	NS	− 0.35	NS
Probing depth	− 0.17	NS	0.51	Sig	0.44	Sig
RBS	-	-	0.09	NS	0.68	Sig

* Pearson's correlation coefficient, NS <0.5, S>0.5, RBS: Random blood sugar.

## DISCUSSION

Periodontal disease process is site specific and has multifactorial origin where periodontal pathogens, host response, genetic, systemic and behavioral risk factors interplay to develop the disease process. Hence, various measures have been taken to include microbial, immunologic, systemic, genetic and behavioral factors, in addition to traditional clinical and radiographic parameters, for assessing patient's periodontal status.

The activity of β-Glucuronidase is associated with severity of inflammation and also with the presence of putative pathogenic flora. This enzyme has been detected in GCF of patients with active site of periodontal disease, thus making β-Glucuronidase as an important biochemical marker for disease activity and for predicting future attachment loss.[3]

Diabetes mellitus encompasses a heterogenous group of disorders with common characteristics of altered glucose tolerance and impaired lipid and carbohydrate metabolism. Diabetes develops from either deficiency in insulin production or an impaired utilization of insulin.

Diabetes is linked to severe periodontal destruction. Uncontrolled diabetic frequently show a combination of inflammatory and degenerative changes ranging from a mild gingivitis to advanced chronic periodontitis with a widened periodontal ligament, exudation from periodontal pockets, and/or multiple lateral periodontal abscesses. Although it may be more severe in nature, the periodontal disease of the diabetic seems to be clinically similar to that found in non-diabetic individuals. This suggests that the diabetic state serves as a predisposing factor which can accelerate periodontal destruction originated by microbial agents.

In the present study, we have attempted to test the hypothesis that ‘diabetics with chronic periodontitis have higher levels of disease activity markers in GCF as compared to non-diabetics with chronic periodontitis’. The probing pocket depth in chronic periodontitis patients with or without diabetes was standardized, so that these parameters did not have any bearing on the level of activity of GCF β-glucuronidase, thus allowing comparisons to be made between β-glucuronidase activity of diabetic and non-diabetic patients. Also, this study was aimed to determine β-glucuronidase activity in host tissues and not from the bacterial origin hence acidic pH was used to detect activity of enzyme.[6]

In this study, plaque index, gingival index and probing pocket depth values were significantly lower in Group I, as compared to Group II and Group III patients. Though there was statistically non-significant difference in plaque index and gingival index in between Group II and Group III patients, the average probing pocket depth was significantly higher in Group III as compared to Group II. These findings are consistent with that of Oliver and Tervonen[5] and Lamster *et al*.[8]

The relationship between oral hygiene to periodontal disease has long been well established in the dental literature. In this study, with respect to oral hygiene (Plaque Index) and gingival status (Gingival index), the values in diabetic and non-diabetic patients with chronic periodontitis (Group III and II) were statistically not significant. However, when compared with Group I patients, these values were significantly higher.

In Group I (periodontally healthy patients), low levels of β-glucuronidase activity could be demonstrated, similar to the findings of Lamster *et al*.[9] In their study they observed that even in periodontally healthy individuals, some amount of subclinical inflammation was always present. Hence, in the present study, the presence of low levels of β-glucuronidase in Group I patients also indicates the presence of subclinical gingival inflammation.

This study demonstrates that there is an increase in GCF β-glucuronidase level in Group II patients i.e. non diabetic with chronic periodontitis (t=6.92, *P* < 0.001), as compared to healthy individuals, showing highly significant correlation between GCF β-glucuronidase level and probing pocket depth. These results are consistent with the findings of Bang *et al.*[9] and Lamster *et al*.[10] These authors observed that β-glucuronidase increases with the development and severity of inflammation resulting into increased probing pocket depth and attachment loss. Also, the higher levels of β-glucuronidase can be predictive of attachment loss on a site specific and whole mouth basis with high levels of sensitivity and specificity.[10]

The present study also demonstrates that there is higher level of GCF β-glucuronidase activity in patients of diabetes with chronic periodontitis as compared to non-diabetic with chronic periodontitis (t=2.09, *P* < 0.05). This finding is in accordance with study by Bacic *et al.*,[11] who showed that periodontal disease is more frequent and severe in diabetics as compared to non-diabetics. Hayden and Bucklay[12] demonstrated that in diabetics with periodonitis, other than impaired glucose metabolism, genetic predisposition plays an important role in the progression of disease.

Shlossman *et al*,[13] stated that diabetes is a risk factor for development of periodontal disease and there is a significant progressive periodontal destruction in diabetic patients. Cutler *et al*,[14] claimed that the increased susceptibility of diabetic patients to periodontal breakdown may be due to an abnormal PMN's function.

It is also observed that there was a significant positive correlation between GCF β-Glucuronidase and RBS levels, in diabetics with chronic periodontitis. This observation is comparable to the findings of Oliver *et al*,[5] who demonstrated increased β-glucuronidase levels in GCF in uncontrolled diabetics. It has been hypothesized that increased GCF β-glucuronidase activity may be due to hyperactivity and increased deregulation of lysosomes of polymorphonuclear leucocytes in diabetes mellitus. Though, Ginwala *et al*,[15] did not find any significant correlation between blood sugar level and β-glucurondiase levels. In their study, salivary and serum β-Glucuronidase levels were assessed rather than the GCF so, their results can not be correlated with the present one.

Another important observation made in the present study was that even with difference in clinical parameters between study groups, periodontitis patients irrespective of their diabetic status, showed an increased periodontal destruction with elevated β-Glucurondiase level than control. This suggests that β-Glucuronidase level can be used as a biochemical marker for chairside diagnostic kit, which can diagnose early phases of disease with reasonable confidence.

## CONCLUSIONS

Following conclusions were drawn from the present study:

1. Periodontitis patients irrespective of their diabetic status, showed increased periodontal destruction with elevated levels of β-Glucuronidase, than in the control group.
2. Diabetic patients had highest level of β-Glucuronidase level and increased severity of periodontal destruction. This confirms the fact that diabetes is a risk factor for periodontal disease.
3. The presence of β-Glucuronidase level in GCF can be used as a biochemical marker for diagnosing the chronic periodontitis cases.
